# Supplementary material for: RSM- and ANN-Based Multifrequency Ultrasonic Extraction of Polyphenol-Rich Sargassum horneri Extracts Exerting Antioxidative Activity via the Regulation of MAPK/Nrf2/HO-1 Machinery
Source: Antioxidants (Basel). 2024 Jun 4;13(6):690. doi: 10.3390/antiox13060690 (PMC11200430; doi:10.3390/antiox13060690)
Supplement: Supplementary file 1 [file antioxidants-13-00690-s001.zip › antioxidants-2979925-supplementary.pdf]

Table 1S. Setting parameters of genetic algorithm used in the model optimization of SH.

**Setting parameters**	**Values**
Scaling function	Rank
Plot function	Best fitness
Crossover function	Constraint dependent
Elite count	Default
Population size	95
Selection function	Stochastic uniform
Nonlinear constraint algorithm	Augmented Lagrangian
Mutation function	Constraint dependent

Table 2S. ANVOA analysis for developed TPC, TFC, DPPH and ABTS models.

(A) ANOVA for Quadratic model

Response 1: TPC

Source	Sum of Squares	df	Mean Square	F-value	p-value	
**Model**	1575.78	14	112.56	12.76	< 0.0001	significant
A-Concentration	695.20	1	695.20	78.80	< 0.0001	
B-Time	83.75	1	83.75	9.49	0.0095	
C-Temperature	148.21	1	148.21	16.80	0.0015	
D-Frequency	6.21	1	6.21	0.7045	0.4177	
AB	6.67	1	6.67	0.7566	0.4015	
AC	12.27	1	12.27	1.39	0.2611	
AD	43.95	1	43.95	4.98	0.0454	
BC	139.44	1	139.44	15.81	0.0018	
BD	24.02	1	24.02	2.72	0.1248	
CD	99.35	1	99.35	11.26	0.0057	
A^2	125.18	1	125.18	14.19	0.0027	
B^2	46.04	1	46.04	5.22	0.0413	
C^2	209.83	1	209.83	23.78	0.0004	
D^2	85.88	1	85.88	9.73	0.0089	
**Residual**	105.87	12	8.82			
Lack of Fit	103.97	10	10.40	10.98	0.0863	not significant
Pure Error	1.89	2	0.9472			
**Cor Total**	1681.65	26				

(B) ANOVA for Quadratic model

Response 2: TFC

Source	Sum of Squares	df	Mean Square	F-value	p-value	
**Model**	3628.07	14	259.15	13.27	< 0.0001	significant
A-Concentration	2156.10	1	2156.10	110.41	< 0.0001	
B-Time	42.35	1	42.35	2.17	0.1666	
C-Temperature	2.50	1	2.50	0.1279	0.7268	
D-Frequency	76.77	1	76.77	3.93	0.0708	
AB	29.54	1	29.54	1.51	0.2423	
AC	1.84	1	1.84	0.0941	0.7643	
AD	35.17	1	35.17	1.80	0.2044	
BC	16.14	1	16.14	0.8264	0.3812	
BD	18.53	1	18.53	0.9489	0.3492	
CD	14.06	1	14.06	0.7202	0.4127	
A^2	519.72	1	519.72	26.61	0.0002	
B^2	22.55	1	22.55	1.15	0.3037	
C^2	159.26	1	159.26	8.16	0.0145	
D^2	14.80	1	14.80	0.7580	0.4011	
**Residual**	234.34	12	19.53			
Lack of Fit	231.28	10	23.13	15.16	0.0634	not significant
Pure Error	3.05	2	1.53			
**Cor Total**	3862.40	26				

Table 2S. ANVOA analysis for developed TPC, TFC, DPPH and ABTS models.

(C) ANOVA for Quadratic model

Response 3: DPPH

Source	Sum of Squares	df	Mean Square	F-value	p-value	
**Model**	586.35	14	41.88	8.77	0.0003	significant
A-Concentration	282.44	1	282.44	59.16	< 0.0001	
B-Time	35.37	1	35.37	7.41	0.0185	
C-Temperature	2.77	1	2.77	0.5806	0.4608	
D-Frequency	16.05	1	16.05	3.36	0.0916	
AB	2.89	1	2.89	0.6048	0.4518	
AC	20.40	1	20.40	4.27	0.0610	
AD	64.18	1	64.18	13.44	0.0032	
BC	58.47	1	58.47	12.25	0.0044	
BD	30.77	1	30.77	6.45	0.0260	
CD	1.38	1	1.38	0.2890	0.6007	
A^2	119.67	1	119.67	25.06	0.0003	
B^2	55.43	1	55.43	11.61	0.0052	
C^2	34.70	1	34.70	7.27	0.0195	
D^2	29.22	1	29.22	6.12	0.0293	
**Residual**	57.29	12	4.77			
Lack of Fit	50.83	10	5.08	1.57	0.4505	not significant
Pure Error	6.47	2	3.23			
**Cor Total**	643.64	26				

(D) ANOVA for Quadratic model

Response 4: ABTS

Source	Sum of Squares	df	Mean Square	F-value	p-value	
**Model**	1573.93	14	112.42	12.29	< 0.0001	significant
A-Concentration	665.52	1	665.52	72.76	< 0.0001	
B-Time	46.99	1	46.99	5.14	0.0427	
C-Temperature	0.1385	1	0.1385	0.0151	0.9041	
D-Frequency	1.36	1	1.36	0.1487	0.7066	
AB	62.31	1	62.31	6.81	0.0228	
AC	0.4818	1	0.4818	0.0527	0.8224	
AD	51.89	1	51.89	5.67	0.0346	
BC	2.27	1	2.27	0.2482	0.6274	
BD	34.86	1	34.86	3.81	0.0746	
CD	0.0228	1	0.0228	0.0025	0.9610	
A^2	342.06	1	342.06	37.39	< 0.0001	
B^2	133.49	1	133.49	14.59	0.0024	
C^2	310.85	1	310.85	33.98	< 0.0001	
D^2	194.70	1	194.70	21.29	0.0006	
**Residual**	109.77	12	9.15			
Lack of Fit	108.28	10	10.83	14.58	0.0658	not significant
Pure Error	1.49	2	0.7426			
**Cor Total**	1683.70	26				

Table 3S. Fit statistics values of independent variables and corresponding target responses.

(A) Fit Statistics value for TPC

**Std. Dev.**	2.97	**R^2**	0.9370
**Mean**	59.22	**Adjusted R^2**	0.8636
**C.V. %**	5.02	**Predicted R^2**	0.6490
		**Adeq Precision**	13.0466

(B) Fit Statistics value for TFC

**Std. Dev.**	4.42	**R^2**	0.9393
**Mean**	33.44	**Adjusted R^2**	0.8685
**C.V. %**	13.22	**Predicted R^2**	0.5857
		**Adeq Precision**	11.8726

(C) Fit Statistics value for DPPH

**Std. Dev.**	2.19	**R^2**	0.9110
**Mean**	23.46	**Adjusted R^2**	0.8071
**C.V. %**	9.31	**Predicted R^2**	0.5095
		**Adeq Precision**	11.0479

(D) Fit Statistics value for ABTS

**Std. Dev.**	3.02	**R^2**	0.9348
**Mean**	32.92	**Adjusted R^2**	0.8587
**C.V. %**	9.19	**Predicted R^2**	0.6034
		**Adeq Precision**	11.6513

Table 4S. Comparison of the prediction abilities of the RSM and ANN models.

Parameters	TPC		TFC		DPPH		ABTS	
RSME	1.98	1.58	2.4	2.11	1.45	0.91	2.01	1.32
R^2	93.70	95.98	93.93	96.87	91.09	96.45	93.48	97.20
AAD (%)	2.82	1.83	8.05	5.21	5.79	2.76	5.24	3.61
SEP (%)	0.12	0.09	0.32	0.23	0.22	0.14	0.22	0.14

Table 5S. RSM and ANN optimized model condition.

Model	Concentration	Time	Temperature	Frequency	TPC	TFC	DPPH	ABTS	Desirability
RSM model	46.281	27.598	33.257	36.978	64.386	23.716	28.780	42.356	1.00
ANN model	48.375	28.764	34.637	36.843	67.510	33.489	27.246	44.456	-

Table 6S. Binding affinity of identified bioactive compounds for keap1 protein (4L7B).

Identified compounds	Binding energy (Kcal/mol)	Identified compounds	Binding energy (Kcal/mol)
Ferulic acid	6.6	2-Hydroxy-2-phenylacetic acid	6.1
Sinapic acid	6.2	Isopropyl 3-(3,4-dihydroxyphenyl)-2-hydroxypropanoate#	6.1
Protocatechuic acid	6.5	Epigallocatechin	8
Caffeic acid	6.2	Epicatechin	8.5
Syringic acid	6.7	Dalbergin	8.3
Salicylic acid	5.6	Catechin	8.4
Cinnamoyl glucose	6.8	Dihydrobiochanin A	8
p-Hydroxybenzaldehyde	5.4	Glycitein 7-O-glucuronide	9.5
Hydroxytyrosol 4-O-glucoside	7.5	Arctigenin	8.8
5-(3′,4′-Dihydroxyphenyl)-valeric acid	6.9	Isohydroxymatairesinol	9.1
5-(3′,5′-Dihydroxyphenyl)-γ-valerolactone 3-O-glucuronide	5.9	Conidendrin	9.1
Quinic acid	5.8	Deoxyschisandrin	7.5
Syringin	7.1	Secoisolariciresinol	7.8
Coumaroylquinic acid	8.5	Carnosol	9
3-Sinapoylquinic acid	7.5	Carnosic acid	8
Hydroxyferulic acid	8.4	Loliolide	6.6
Caffeoyl tartaric acid	7.6	Isololiolide	6.5
Vanillic acid 4-sulfate	6.7	Sargahydroquinoic acid	8
Vanillic acid	6.4	Sargaquinoic acid	9.2
3,4-Dihydroxyphenylglycol	7.6	Dihydroactinidiolide	6.6
Mojabanchromanol	7.2	Humulene epoxide II	7.8
Fallahydroquinone	6.9	(-)-Isoamijiol	8.3
Fallaquinone	6.8	3,5-Dihydroxy-6,7-megastigmadien-9-one	7.3
1-H-indol-6-Carbaldehde	5.9	Phloroglucinol	6.7
Trifuhalol-A	8.8	Fucophlorethol	6.7
1-O-(11-hexadecenoly)-3-O-(6′-sulpho-a-D-quinovopyranosyl)glycerol	6.9	Eckol	9.1
1-O-Hexadecanoyl-3-O-(60 -sulfo-a-Dquinovopyranosyl) glycerol	7.7	Dioxinodehydroeckol	8.8
		Dibenzodioxin-1,3,6,8-tetraol#	7.9
